# Zinc Deficiency in Chronic Kidney Disease and Hemodialysis: Insights from Basic Research to Clinical Implications

**DOI:** 10.3390/nu17132191

**Published:** 2025-06-30

**Authors:** Shinya Nakatani, Tomoaki Morioka, Fumiyuki Morioka, Katsuhito Mori, Masanori Emoto

**Affiliations:** 1 Department of Metabolism, Endocrinology and Molecular Medicine, Osaka Metropolitan University Graduate School of Medicine, 1-4-3 Asahi-machi, Abeno-ku, Osaka 545-8585, Japan; 2 Department of Nephrology, Osaka Metropolitan University Graduate School of Medicine, Osaka 545-8585, Japan

**Keywords:** zinc, hemodialysis, chronic kidney disease

## Abstract

Zinc is an essential trace element involved in diverse physiological processes in humans. Zinc deficiency is common in patients with chronic kidney disease (CKD), including those undergoing hemodialysis. This narrative review synthesizes both experimental and clinical findings on zinc status in CKD patients. Literature was primarily retrieved from PubMed using the keywords “zinc” AND (“CKD” OR “hemodialysis”) AND at least one of the following: “cardiovascular disease (CVD)”, “vascular calcification”, “anemia”, “blood pressure”, OR “infection”. In vitro, studies have shown that zinc suppressed phosphate-induced vascular calcification while zinc deficiency directly promoted calcification. Clinically, serum zinc levels were positively correlated with calcification propensity in patients with CKD. In vivo zinc deficiency has been implicated in elevated blood pressure, Moreover, zinc supplementation enhanced erythropoiesis and improved responsiveness to erythropoiesis-stimulating agents in both animal models and humans. We recently reported that low serum zinc levels are associated with increased mortality in hemodialysis patients with hypoalbuminemia. Previous randomized controlled trials (RCTs) suggest a daily dose of approximately 45 mg of zinc for 2 months mitigates inflammation, oxidative stress, and malnutrition in patients undergoing hemodialysis. Emerging evidence suggests that vascular calcification, hypertension, and renal anemia are newly recognized features of zinc deficiency and are established risk factors for CKD progression, CVD, and mortality. However, the impact of zinc supplementation on these clinical outcomes remains inconclusive. Further RCTs are required to establish zinc supplementation as an effective therapeutic strategy for improving various outcomes in patients with CKD including hemodialysis.

## 1. Introduction

**Figure 1:**
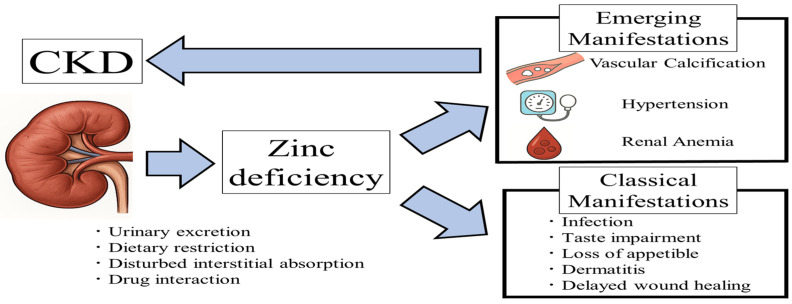
Associations between CKD and clinical manifestations of zinc deficiency.

Zinc is an essential trace element and the second most abundant divalent cation in the body [1]. Zinc deficiency is a global issue, affecting over two billion people [2]. In patients with CKD, including those undergoing hemodialysis, zinc deficiency results from multiple factors, including inadequate dietary intake due to uremia-related anorexia and dietary restrictions, impaired absorption caused by certain medications, increased urinary zinc excretion associated with CKD progression, and zinc removal during dialysis [3,4,5]. Additionally, in a rodent model of CKD induced by 5/6 nephrectomy, elevated intestinal luminal phosphate levels caused by hyperphosphatemia and/or dietary phosphate intake impaired intestinal zinc absorption, leading to zinc deficiency [6] (Figure 1). Zinc concentrations are categorized according to the guidelines for zinc deficiency by the Japanese Society of Clinical Nutrition as follows: deficiency (<60 μg/dL), marginal deficiency (≥60 to <80 μg/dL), and normal (≥80 μg/dL) [7]. Marginal zinc deficiency is commonly observed in patients with pre-dialysis CKD, whereas zinc deficiency is predominant among those undergoing hemodialysis [8]. We have previously demonstrated a reduced blood zinc levels in patients with CKD including those undergoing hemodialysis [8]. A recent meta-analysis, which included 42 studies with patients with CKD (*n* = 460), hemodialysis (*n* = 2047), and healthy controls (*n* = 1657) showed that blood zinc levels in patients with CKD and hemodialysis were significantly lower than those in healthy controls (mean difference = −22.86 μg/dL, 95% CI −33.25 to −12.46; mean difference = −13.64 μg/dL, 95% CI −21.47 to −53.80, respectively) [9].

The daily requirement of zinc for adults throughout the world vary and are as follows: WHO—for females 3.0–9.8 mg/d, and for males 4.2–14.0 mg/day; the European Food Safety Agency—7.5–12.7 mg/day for females and 9.4–16.3 mg/day for males, the amount depending on the phytate in the diet [10]; and dietary intake of zinc in Japan for adults—9–10 mg/day for males and 7–8 mg/day for females [11], although intake is insufficient in 60–70% of both genders aged over 20 years [7]. Additionally, zinc intake was found to be lower in patients with advanced stage CKD as compared with those without advanced stage CKD who participated in the National Health and Nutrition Examination Survey conducted in the United States [12]. Patients with zinc deficiency including CKD are initially recommended to consume zinc-enriched foods, e.g., oysters, scallops, tofu, rice, and fermented soybeans (Natoo) [7].

When dietary therapy is insufficient, the Practical Guideline recommends administering zinc to patients with zinc deficiency at a dosage of 50–100 mg/day for adults [7]. Organic compounds such as zinc histidinate, zinc gluconate and zinc orotate show a comparatively better tolerability than inorganic zinc sulfate and zinc chloride [13]. A total of 20 RCTs have investigated zinc supplementation in patients undergoing hemodialysis (Table 1) [14,15,16,17,18,19]. These findings suggest that a daily dose of approximately 45 mg of zinc for 2 months resulted in increased serum zinc levels, SOD activity, and dietary protein intake, as well as reduced levels of C-reactive protein (CRP) and malondialdehyde [18]. Another meta-analysis reported that zinc supplementation led to increases in body weight and/or body mass index (BMI) in patients undergoing hemodialysis [20].

In patients with CKD, including those undergoing hemodialysis, zinc deficiency arises from multiple factors. In addition to classical manifestations of zinc deficiency, emerging evidence highlights vascular calcification, hypertension, and renal anemia as newly recognized features of zinc deficiency. These emerging manifestations have been identified as the risk factors for CKD progression. Abbreviations: CKD, chronic kidney disease.

Zinc serves as an essential cofactor for the activity of approximately 300 zinc-containing enzymes, including DNA polymerase, RNA polymerase, alcohol dehydrogenase, carbonic anhydrase, and superoxide dismutase (SOD) [36,37]. Bioinformatics research suggests that approximately 2800 human proteins may function as zinc-binding proteins [38]. In addition to traditional symptoms of zinc deficiency such as taste impairment [39], anorexia and loss of appetite [40], dermatitis [41], delayed wound healing [42], and infection [43], other clinical manifestations including vascular calcification, hypertension, and renal anemia, have been increasingly recognized in patients with CKD [44] (Figure 1). These emerging features of zinc deficiency are associated with adverse outcomes, including progressive kidney dysfunction, cardiovascular disease (CVD) events, and increased mortality. Regarding other trace elements, including magnesium and selenium, low serum levels of these elements have been consistently associated with CKD progression in pre-dialysis patients [45,46,47], as well as with all-cause mortality in patients undergoing hemodialysis [48,49]. However, evidence regarding the association between serum zinc levels and these clinical outcomes remains relatively limited.

The purpose of this review is therefore to clarify the clinical implications of zinc deficiency in patients with CKD, particularly those undergoing hemodialysis, and to provide a comprehensive overview of experimental and clinical studies investigating zinc supplementation strategies. This narrative review synthesizes both experimental and clinical findings on zinc status in CKD patients. Literature was primarily retrieved from PubMed using the keywords “zinc” AND (“CKD” OR “hemodialysis”) AND at least one of the following: “CVD” “vascular calcification”, “anemia”, “blood pressure”, OR “infection”. These studies have investigated various dosages and frequencies, as well as the potential effects of zinc supplementation in CKD. Through this review, we aim to emphasize the need for well-designed future clinical trials to confirm the therapeutic value of zinc supplementation in this population.

## 2. Zinc and Calcification

### 2.1. Vasculature Smooth Muscle Cells

Phosphate was first identified as essential for calcification in vascular smooth muscle cells (VSMCs) more than 25 years ago [50]. Since then, numerous studies have investigated potential factors that could mitigate phosphate-induced calcification. Among these, magnesium [51,52] and zinc [53,54] have been identified as playing critical roles in inhibiting phosphate-induced calcification in VSMCs. Regarding zinc, zinc sulfate was shown to blunt phosphate-induced calcification and decrease messenger RNA expression of osteogenic markers. The inhibition of cellular calcification by zinc is partly mediated through the zinc-sensing receptor GPR39 [54,55,56]. In the experiment using primary human aortic VSMCs and klotho-hypomorphic, subtotal nephrectomy, and cholecalciferol-overload mouse calcification models, zinc activated tumor necrosis factor α-induced protein 3 (TNFAIP3), which inhibits nuclear factor kappa B (NF-κB) activation and osteo-/chondrogenic reprogramming, thereby suppressing phosphate-induced calcification in VSMCs [54]. Additionally, zinc sulfate inhibited VSMCs calcification induced by high glucose levels through the suppression of NF-κB activation [56].

Interestingly, zinc deficiency has also been reported to directly promote calcification. In rat aortic VSMCs under zinc-deficient conditions, the expression of Pit1, a sodium-dependent phosphate transporter that facilitates phosphate uptake and regulates calcification was significantly upregulated. Subsequently, treatment with a Pit1 inhibitor ameliorated these calcifications in rat aortic VSMCs [57]. Furthermore, excessive zinc specifically inhibited the in vitro osteogenic differentiation of bone marrow-derived mesenchymal stem cells (BMSCs) by regulating phosphate uptake. This resulted in the downregulation of key osteo-chondrogenic transcription factors Runt-related transcription factor 2 (RUNX2) and SRY-box transcription factor 9 (SOX9), along with their target genes osteocalcin (OCN) and alkaline phosphatase (ALP) [58]. These in vitro findings suggest that zinc may be a critical microelement for inhibiting phosphate-induced calcification.

### 2.2. Serum Calcification Propensity: T50

In serum, precipitation of supersaturated calcium and phosphate is prevented by the formation of amorphous calciprotein particles (CPPs) [59,60]. CPPs is a colloidal mineral–protein complex mainly composed of solid-phase phosphate, calcium, and serum protein fetuin-A [61,62]. Small and spherical CPPs (primary CPPs) which contain amorphous calcium phosphate clusters, spontaneously convert into aggregate and transform into larger and irregular-shaped ones (secondary CPPs). These secondary CPPs contain crystallized calcium phosphate and exhibit cytotoxic and proinflammatory properties, promoting inflammation, atherosclerosis, and vascular calcification [63,64].

Serum calcification propensity, referred to as T50 [65] is biomarker of vascular calcification [66]. T50 is measured using nephelometry, which detects laser light scatter in turbid samples. The assay relies on the formation of primary CPPs by addition of exogenous calcium and phosphate to a human serum sample. The T50 value represents the time required for 50% of primary CPPs to convert into secondary CPPs. A shorter T50 indicates a higher propensity for calciprotein crystallization, reflecting increased calcification stress [65]. Notably, a shorter T50 has been associated with arterial calcification [67] and a higher risk for CVD events and all-cause mortality in patients with CKD including hemodialysis [66,67,68,69,70,71]. Therefore, T50 is considered as a surrogate marker of calcification stress and is closely associated with CVD risk in those patients.

A positive correlation between serum zinc levels and T50 has been observed in both individuals with normal kidney function (r = 0.328, *p* = 0.013) and patients with CKD (r = 0.265, *p* = 0.002) [54]. Similarly, our cross-sectional study involving 132 patients with type 2 diabetes mellitus and varying kidney function also reported a positive correlation (r = 0.219, *p* = 0.012) [72], in which serum T50 was determined in duplicate over a period of 600 min per measurement using a nephelometer (Nephelostar Plus®, BMG Labtech, Saitama, Japan), and the measurement results were analyzed using the MARS software (BMG Labtech, Saitama, Japan). We also demonstrated that addition of a physiological concentration of exogenous zinc chloride significantly increased serum T50 [72]. Furthermore, a recent study found that higher serum zinc levels were associated with a longer T50 and a lower hydrodynamic radius of secondary CPPs in patients with CKD including those undergoing hemodialysis (r = 0.488, *p* < 0.001) [73]. When 100 µM zinc (corresponding to 6538 µg/L) was added to serum samples from healthy volunteers, T50 was prolonged. In contrast, a much higher concentration of 1 mM was required to reduce the hydrodynamic radius of secondary CPPs, suggesting that the direct effect of zinc may have a greater impact on T50 than on the size of secondary CPPs [40]. Even in polyethylene glycol hydrogels, not in serum, zinc was shown to inhibit the transformation of amorphous calcium phosphate (ACP) into hydroxyapatite [74]. In additive-free composite, ACP transformed into brushite within minutes. In contrast, in the presence of zinc, zinc-doped ACP was very stable and did not show any signs of crystallization for up to 20 days. In ACP, zinc ion readily substitutes calcium, suppressing crystallization by decreasing solubility [75]. It is thus likely that zinc suppresses the transformation from amorphous primary CPPs into secondary CPPs, containing crystalline hydroxyapatite, in serum. Although the precise mechanism by which zinc prolongs T50 remains unclear, its inhibitory effect on hydroxyapatite formation in vitro [76] may contribute to this phenomenon.

### 2.3. Vasculature Change

Coronary artery calcification (CAC) is highly prevalent in patients with CKD [77]. The CAC score has been strongly and significantly associated with an increased risk of subsequent CVD and all-cause mortality, as evidenced by findings from the Chronic Renal Insufficiency Cohort study [78]. Regarding zinc and CAC scores, whole blood zinc levels in patients with CKD were found to be significantly and negatively correlated (r = −0.601, *p* < 0.001) [79].

Abdominal aorta calcification (AAC) is also common in CKD and is recognized as an independent predictor of cardiovascular mortality in both the general population and patients with CKD [80,81]. Serum zinc levels were significantly and negatively associated with AAC score in patients undergoing hemodialysis (r = −0.43, *p* = 0.005) [82]. Among non-institutionalized CKD patients in the United States, whose mean dietary zinc intake was 10.5 mg/day, each 1 mg/day increase in zinc intake was associated with a reduced risk of severe AAC after adjustment for age, gender, and ethnicity [12].

Carotid intima-media thickness (CIMT) is a valuable marker of subclinical atherosclerosis [83]. In a study of middle-aged and older adults, individuals with lower zinc intake exhibited greater CIMT compared to those with higher intake. [84]. Similarly, serum zinc levels were associated with CIMT in patients undergoing hemodialysis (r = −0.70, *p* < 0.01) [85].

The pulse wave velocity (PWV) of the aorta is a key indicator of arterial wall stiffness and a predictor of CVD mortality and all-cause mortality in patients undergoing hemodialysis [86]. In non-diabetic hemodialysis patients, zinc levels were identified as independent predictors of brachial-ankle PWV (β = −9.85, *p* = 0.047) after adjustment for hypoalbuminemia and CRP levels [87].

The effect of zinc supplementation on vascular changes in humans has not been reported. However, in vivo studies have shown that zinc sulfate ameliorated vascular calcification in hyperphosphatemic kl/kl mice. This was demonstrated through aortic Alizarin Red staining, von Kossa staining of thoracic aorta sections, and quantification of calcium content in the aortic arch [54].

**Figure 2:**
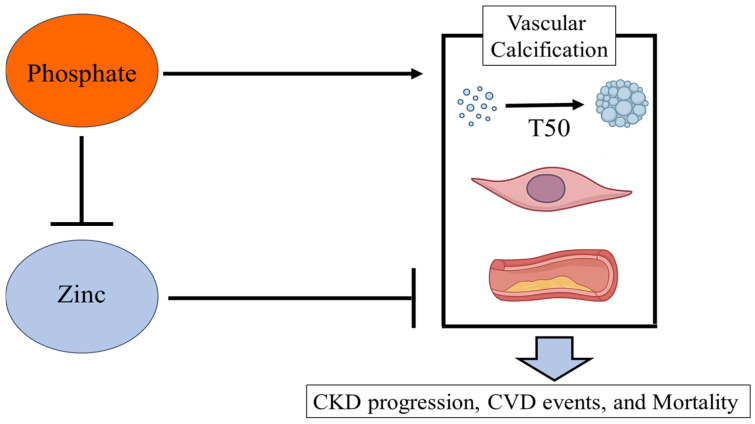
Associations between zinc and phosphate in vascular calcification and clinical outcomes.

Since zinc deficiency is common in patients with CKD, the inhibitory effects of zinc on hydroxyapatite formation (i.e., prolongation of T50) and the suppression of calcification-related transcription factors may be impaired in patients with CKD. Findings from basic research on zinc in relation to VSMCs and T50, along with clinical studies on zinc and markers of vascular calcification and atherosclerosis, suggest that zinc could help suppress the progression of CKD, CVD events, and mortality by preventing phosphate-induced calcification (Figure 2). Further clinical trials are warranted to determine whether zinc supplementation can improve T50 and/or attenuate vascular calcification in patients with CKD and those undergoing hemodialysis.

Phosphate promotes the transition from primary calciprotein CPPs to secondary CPPs, a process that plays a pivotal role in vascular calcification and contributes to the progression of CKD, CVD events, and mortality. Experimental studies have demonstrated that zinc suppresses phosphate-induced vascular calcification. Conversely, hyperphosphatemia and excessive dietary phosphate intake can impair intestinal zinc absorption, potentially leading to zinc deficiency. Abbreviations: CKD, chronic kidney disease; CPPs, calciprotein particles; CVD, cardiovascular disease.

## 3. Zinc and Blood Pressure

A meta-analysis revealed an association between serum zinc levels and increased blood pressure across various region including Africa, North America, Asia, and Europe [88]. In hypertension model rats, dietary zinc restriction exacerbated systolic blood pressure [89], whereas zinc supplementation attenuated blood pressure response [90]. Although the precise mechanisms by which zinc contributes to blood pressure control remain uncertain, it has been suggested that zinc may enhance the activity of nitric oxide synthase (NOS), leading to increased nitric oxide (NO) production and vasodilation [91]. It has been proposed that zinc may also enhance the activity of superoxide scavenger enzymes, thereby facilitating the removal of superoxide radicals [92,93]. Therefore, zinc deficiency may impair downstream actions of endothelial NO, leading to insufficient vasodilation. Additionally, zinc has recently been shown to play a regulatory role in renal sodium reabsorption in the distal nephron [94]. While approximately 70% of sodium is reabsorbed in the proximal tubules, fine-tuning of renal sodium handling occurs in the distal nephron. In C57BL/6 mice, zinc deficiency induced hypertension because of impaired renal sodium excretion [94]. Interestingly, under high-salt conditions, zinc deficiency may significantly affect urinary sodium reabsorption by altering renal tubular epithelial sodium transporters, thereby contributing to hypertension in C57BL/6 mice [95]. Future studies are needed to explore the potential of zinc supplementation as a strategy to prevent blood pressure elevation in patients with CKD and those undergoing hemodialysis.

## 4. Zinc and Anemia

Zinc deficiency may contribute to the development of anemia by increasing the fragility of erythrocyte membranes, decreasing iron availability and impairing erythropoiesis [96,97]. The fragility of erythrocyte membranes is thought to result from a reduction in sulfhydryl groups within membrane-bound Na^+^/K^+^-ATPase and Ca^2+^-ATPase enzymes, which weakens membrane integrity and renders red blood cells more susceptible to hemolysis [98]. The differentiation and proliferation of erythroblasts critically require the zinc finger transcription factor GATA-1. Zinc deficiency impairs GATA-1 function, thereby disrupting erythroid maturation and leading to anemia [99]. Several studies using animal models have also indicated that zinc deficiency impairs erythropoiesis, highlighting the essential role of zinc in this process [97,100,101]. Zinc supplementation has been shown to stimulate erythropoiesis in rats with phenylhydrazine-induced anemia and those with 5/6 nephrectomy-induced anemia, as well as to promote red blood cell formation in the bone marrow [102]. In patients undergoing hemodialysis, zinc deficiency has been associated with reduced responsiveness to erythropoiesis-stimulating agent (ESA) [103]. In addition, the mechanical stimulation of hemodialysis may cause the rupture of red blood cells leading to anemia in patients undergoing hemodialysis [96]. Supplementation with polaprezinc, a zinc-containing compound (zinc L-carnosine), at a dose of 34 mg of elemental zinc per day has been found to improve anemia and decrease ESA doses from 7125 ± 1196 IU/week to 5427 ± 2860 IU/week [104]. Additionally, administration of polaprezinc for 12 months has been shown to reduce both the ESA dosage and the erythropoietin responsiveness index, which is calculated as weekly ESA dose (units) divided by dry weight (kg) and hemoglobin level (g/dL), in patients undergoing hemodialysis. The ESA dosage decreased from approximately 110 to 80 U/kg/week, and the erythropoietin responsiveness index decreased from 11 to 8, respectively [21]. Further research is needed to evaluate whether zinc supplementation can improve responsiveness to ESA and alleviate renal anemia in patients with CKD.

Hypoxia-inducible factor stabilizers (HIF stabilizers), also known as HIF prolyl hydroxylase inhibitors (HIF-PHIs), are used to treat anemia in CKD by stimulating the production of endogenous erythropoietin and improving iron metabolism [105]. However, recent findings have highlighted that HIF-PHIs also play a pivotal role in vascular calcification in VSMCs [106,107]. Specifically, HIF-1α may accelerate calcium deposition by upregulating the expression of the master regulator of osteogenic differentiation [106]. FG4592, an orally bioavailable PHI, promotes phosphate uptake in VSMCs and phosphate-induced loss of smooth muscle cell markers (ACTA-2, MYH11, SM22a) and enhances osteochondrogenic gene expression (Msx-2, BMP-2, Sp7). Zinc inhibits FG4592-aggravated calcification caused by high phosphate by maintaining the VSMC phenotype, decreasing phosphate uptake, and lowering osteochondrogenic gene expression and levels of PDK4, as well as preserving Runx2 phosphorylation and cell variability [53]. Additionally, in mice with CKD fed an adenine and high-phosphate diet, treatment with a HIF-PHI was found to accelerate aortic calcification, as assessed using Osteosense dye [108]. To date, no studies have reported such effects of HIF-PHI treatment on vascular calcification in humans. However, caution is warranted when administering HIF-PHIs to patients with CKD, given the potential risk of promoting vascular calcification [109]. Notably, zinc inhibits FG4592-aggravated calcification caused by high phosphate by maintaining the VSMC phenotype, decreasing phosphate uptake, and lowering osteochondrogenic gene expression and levels of PDK4, as well as preserving Runx2 phosphorylation and cell variability [53].

Although it may be challenging to investigate whether HIF-PHIs promote vascular calcification and whether zinc supplementation could mitigate this effect in CKD patients with advanced vascular calcification, such clinical trials remain essential. They could provide valuable insights into developing novel strategies for managing renal anemia while accounting for vascular insights into developing novel strategies for managing renal anemia while accounting for vascular calcification risks.

## 5. Zinc and Other Diseases

### 5.1. Diabetes

Zinc deficiency constitutes a significant risk factor for diabetes, given its essential role as a cofactor in the synthesis, storage, and secretion of insulin by the pancreas [110]. Zinc deficiency may compromise pancreatic insulin synthesis and secretion, and diminish glucose uptake by peripheral tissues, thereby promoting the development of insulin resistance, a defining feature of diabetes mellitus [111]. A recent meta-analysis demonstrated that zinc supplementation significantly reduced fasting blood glucose, glycosylated hemoglobin (HbA1c), serum insulin, and insulin resistance as estimated by the homeostasis model assessment (HOMA-IR) [112].

Several experimental models have indicated that zinc supplementation upregulates metallothionein and nuclear factor erythroid 2–related factor 2 (Nrf2), which may represent key mechanisms linking zinc deficiency to renal function decline in diabetes [113,114]. Additionally, several short-term RCTs in patients with type 2 diabetes have shown that zinc supplementation can reduce urinary microalbumin levels [115,116,117]. However, its effect on kidney function remains controversial, possibly due to the preserved kidney function of participants and the relatively short duration of zinc supplementation in these previous RCTs. Further studies are needed to evaluate the reno-protective effects of zinc supplementation on massive proteinuria and kidney function in patients with advanced stages of CKD with diabetes.

### 5.2. Infection

Zinc deficiency has been reported to impair immunity [118], as it plays a crucial role in regulating the immune response, particularly T-cell–mediated functions [119]. There is substantial evidence linking zinc deficiency to several infectious disease, including malaria, HIV, tuberculosis, measles, and respiratory infections such as COVID-19 pneumonia [120]. Among elderly individuals in nursing homes, those with low serum zinc levels (<70 μg/dL) had a significantly higher incidence and longer duration of pneumoniae [20], as well as all-cause mortality during 1-year follow-up [121]. In patients with CKD stage 5, low serum zinc levels (<50 μg/dL) were associated with a higher risk of infection-related hospitalization [122]. Furthermore, in patients undergoing hemodialysis or peritoneal dialysis, each 1 mg/dL decrease in serum zinc level was associated with a 2.0% increased risk of hospitalization for infection [43].

Zinc supplementation at 30 mg/day for 3 months for nursing home elderly (aged ≥65 y) enhanced T cell function measured by increased anti-CD3/CD28 and phytohemagglutinin-stimulated T cell proliferation [123]. Moreover, zinc supplementation has been shown to reduce the risk of infection in the elderly [124]. A meta-analysis of six randomized controlled trials involving 2216 patients with severe pneumonia suggested that zinc supplementation as an adjunct to standard treatment is effective in reducing mortality associated with severe pneumonia [125]. Systematic reviews and meta-analyses of RCTs in patients undergoing hemodialysis have shown that approximately 45 mg of zinc for 2 months zinc supplementation significantly reduces CRP and malondialdehyde, while enhancing SOD activity [18,126]. These findings suggest that zinc supplementation may play a crucial role in increasing serum zinc levels, enhancing immune function, and preventing infectious diseases such as pneumonia in patients with CKD, including those undergoing hemodialysis.

### 5.3. Cardiac Diastolic Dysfunction

Diastolic dysfunction is associated with adverse outcomes including CVD mortality and major CVD events in patients undergoing hemodialysis [127]. In addition to traditional risk factors, such as older age, diabetes, and hypertension, zinc deficiency-related factors including oxidative stress, malnutrition, and inflammation have also been shown to contribute to impaired myocardial relaxation and ventricular stiffness [128,129]. In patients not requiring kidney replacement therapy, a recent meta-analysis of 27 case–control studies revealed that individuals with idiopathic dilated cardiomyopathy had significantly lower serum zinc levels, whereas no such association was observed in those with ischemic cardiomyopathy [130]. In patients undergoing hemodialysis, lower serum zinc levels were significantly associated with an increased left atrial volume index > 34 mL/m^2^ and E/e’ ratio > 15 [131]. Although the exact mechanism regarding zinc deficiency and diastolic dysfunction remain unclear, current findings suggest that zinc deficiency may play a contributory role in the development of diastolic dysfunction. Further studies are needed to determine whether zinc supplementation can mitigate cardiac dysfunction in patients undergoing hemodialysis.

## 6. Zinc and Progression of Kidney Disease

Serum zinc levels vary significantly across different stages of CKD, with a notable decrease observed in individuals with late-stage CKD [132]. In a cohort of 194 patients with CKD stages 1–4, more advanced CKD stages were independently associated with lower serum zinc levels, even after adjustment for age, sex, smoking status, educational attainment, diabetes, hypertension, and BMI (*p* for trend = 0.002) [133].

Previous observational study in patients with CKD investigating the relationship between zinc levels and kidney function have shown that individuals in the low-zinc group had a significantly increased risk of experiencing the primary outcome, which included progression to end-stage kidney disease or death, with a hazard ratio of 1.81 (95% confidence interval: 1.02–3.24) [134]. Additionally, low plasma zinc levels were correlated with a greater decline in kidney function (r = 0.185, *p* = 0.023) [4]. Recent basic research has examined whether zinc deficiency itself causes pathological changes [135]. In zinc-deficient rats, glomerulosclerosis and interstitial fibrosis along with increased urinary microalbumin and serum creatinine levels were observed. Zinc deficiency may trigger the activation of reactive oxygen species (ROS) and mitochondrial dysfunction, leading to glomerulosclerosis and interstitial fibrosis, respectively. Notably, zinc supplementation significantly ameliorated these pathological alterations [135]. Future clinical trials are needed to evaluate whether zinc supplementation can mitigate kidney function decline in patients with CKD.

IgA nephropathy and nephrotic syndrome are clinically significant causes of CKD. In IgA nephropathy-prone gddY mice, a high-zinc diet resulted in significantly lower mesangial IgA deposition, serum IgA levels, and urinary protein levels compared to a normal-zinc die [136]. Dietary zinc levels may influence the immune process of nephritogenic IgA production by modulating dendritic cell activity, particularly via the Toll-like receptor 4 pathway [136].

Although the precise mechanisms remain unclear, accumulating evidence has highlighted an association between serum zinc levels and the risk of relapse in children with steroid-sensitive nephrotic syndrome, as reported in a recent systematic review [137]. This review demonstrated a significant relationship between reduced serum zinc levels and disease severity. Moreover, zinc supplementation was suggested to support sustained remission and reduce relapse rates [137]. Given the potential efficacy of zinc supplementation in achieving remission in patients with IgA nephropathy and nephrotic syndrome, further clinical studies are warranted.

## 7. Zinc and CVD Events

Epidemiological studies examining the relationship between zinc levels and the risk of CVD events were summarized in a previous review [138], which demonstrated that low serum zinc levels are associated with an increased risk of CVD events. Another systematic review of prospective cohort studies investigating the association between zinc status and CVD events found that higher serum zinc levels were associated with a lower risk of CVD events [139], particularly in patients referred for coronary angiography [140] and patients with type 2 diabetes mellitus [141]. Evidence regarding this topic in patients with CKD is very limited. In a study of 170 CKD patients followed for 40 months, during which 59 CVD events occurred, a Kaplan–Meier analysis with a log-rank test revealed that patients in the lower zinc level group had a higher risk of CVD occurrence. Furthermore, univariate Cox hazard regression analyses demonstrated that zinc levels were significantly associated with a reduced risk of CVD events (hazard ratio of 0.97 (95% confidence interval: 0.04–0.99)). However, this association was no longer significant in multivariate Cox regression analyses [79]. Regarding hemodialysis patients, a longitudinal study involving 42 incident hemodialysis patients followed for 2.5 years, during which 20 CVD events occurred, found no significant association between lower zinc levels and an increased risk of CVD [5]. The relatively small sample size of this study represents a key limitation. Of interest, the association between serum concentrations of other trace elements, such as magnesium and selenium, and CVD events in patients undergoing hemodialysis has also not been clearly established. Further research involving larger cohorts is needed to more clearly elucidate the relationship between trace element levels, including serum zinc, and CVD in patients with CKD and those undergoing hemodialysis.

## 8. Zinc and Mortality in Patients with Hemodialysis

To date, no studies have reported a possible association between serum zinc levels and mortality in patients with pre-dialysis CKD. In contrast, the association between serum zinc levels and mortality in patients undergoing hemodialysis remains controversial (Table 2). A study using a backward stepwise Cox analysis identified older age, hypoalbuminemia, and zinc deficiency as independent predictors of mortality in a cohort of 111 patients on maintenance dialysis, including both hemodialysis and peritoneal dialysis [43]. In patients with incident hemodialysis (*n* = 142), the association between serum zinc levels and all-cause mortality was not significant after adjustment for potential confounders including serum albumin and CRP [5]. Prevalent patients on maintenance hemodialysis (*n* = 61) showed no significant association of serum zinc with mortality [142]. The sample sizes of the three studies were relatively small, with the number of deaths ranging from 11 to 15, which limited the ability to perform sufficient statistical adjustments and draw solid conclusions. In a larger study involving incident hemodialysis patients (*n* = 1278), multivariable-adjusted analysis using a forward stepwise approach by which covariates were selected from many potential confounders including serum albumin showed that lower zinc levels were not associated with an increased risk of death [49]. The inconsistencies in the findings of previous studies may be attributable, at least in part, to the influence of confounding factors such as serum albumin and CRP. Notably, approximately 60–80% of circulating zinc is bound to albumin in the serum [143], highlighting the importance of accounting for this factor in statistical analyses.

Recently, we have investigated the potential association between serum zinc levels and all-cause mortality in a prospective cohort of 1662 prevalent patients undergoing maintenance hemodialysis [144]. Although the serum zinc level was a significant predictor of mortality in a model adjusted for 17 covariates excluding serum albumin, it was not an independent predictor of mortality when the serum albumin level was included. To address serum albumin as a stratification variable rather than a confounder, the cohort was stratified into two groups based on the median serum albumin level (3.7 g/dL). Among patients with lower serum albumin levels, the risk of death was significantly higher in those with lower zinc levels (≤68 µg/dL) compared to their higher zinc counterparts. In contrast, no significant difference in mortality risk was observed between zinc levels among patients with higher serum albumin levels [144]. Our findings indicate that a lower serum zinc level could be a significant factor associated with a higher risk of mortality in patients on maintenance hemodialysis, particularly in those with low serum albumin levels.

Regarding zinc intake, a prospective cohort study of 582 patients undergoing hemodialysis demonstrated a higher mortality risk in those with zinc intake below the recommended values (8 mg per day for women and 10 mg per day for men), even after adjusting for age, diabetes status, gender, dialysis vintage, albumin levels, lean tissue index, energy intake per kilogram, and physical activity levels [145]. RCTs are needed to determine whether zinc supplementation or increased dietary intake could reduce mortality in patients undergoing hemodialysis, particularly those with lower serum albumin levels.

## 9. Clinical Consideration in Zinc Supplementation

Based on the previously presented RCTs, zinc supplementation exceeding 45 mg/day may be necessary to increase serum zinc levels in patients undergoing hemodialysis [8]. However, careful consideration is required for zinc supplementation, as it may impair intestinal copper absorption, potentially leading to copper deficiency and pancytopenia [103]. A previous RCT comparing zinc acetate hydrate (containing 50 mg of zinc) with polaprezinc (containing 34 mg of zinc) showed that serum zinc levels were significantly increased in hemodialysis patients treated with zinc acetate hydrate. However, serum copper levels were significantly lower in the zinc acetate hydrate group compared to the polaprezinc group [16]. These findings suggest that monitoring serum zinc and copper levels is essential during zinc supplementation therapy to avoid trace element imbalances and pancytopenia.

## 10. Future Clinical Trials

To date, several important questions regarding zinc intervention in patients with CKD and those undergoing hemodialysis remain unanswered: (1) the serum zinc levels at which intervention should be initiated; (2) the optimal method of zinc administration (e.g., dietary modification, supplementation, oral formulations, or incorporation into dialysis solutions) and the appropriate duration of treatment; (3) the specific clinical outcomes expected to improve with zinc intervention (e.g., anemia, hypertension, infections, CKD progression, cardiovascular events, or mortality); and (4) the cost-effectiveness of zinc intervention strategies. Although current studies provide evidence to support the role of zinc in mitigating various complications related to CKD, robust clinical trial data remains limited. Most existing RCTs have focused on surrogate endpoints such as serum zinc levels, oxidative stress, and nutritional status, rather than hard clinical outcomes such as CKD progression, CVD events, or mortality. Consequently, the therapeutic potential of zinc supplementation in improving long-term outcomes in patients with CKD, including those undergoing hemodialysis, remains to be conclusively established. Large-scale, well-designed observational studies using target trial emulation or RCTs are needed to determine the clinical efficacy, optimal dosage, safety, and long-term benefits of zinc supplementation in this population.

## 11. Conclusions

Zinc deficiency is common in patients with CKD, including those undergoing hemodialysis. In addition to classical manifestations of zinc deficiency, emerging evidence highlights vascular calcification, hypertension, and renal anemia as newly recognized features of zinc deficiency. These emerging manifestations have been identified as the risk factors for CKD progression, CVD events, and mortality. Experimental studies have demonstrated the antagonistic effects of zinc on hypertension, renal anemia, infections, and phosphate-induced vascular calcification.

The important limitation of this review is the marked heterogeneity among the included studies. The reviewed evidence encompasses a wide range of study designs, zinc formulations, dosages, treatment durations, and patient populations, including different stages of CKD and dialysis modalities (hemodialysis, peritoneal dialysis). These variations pose significant challenges in synthesizing the findings into unified conclusions and may limit both the generalizability and clinical applicability of the results. Nonetheless, this review provides a meaningful overview of the current state of evidence, which may serve as a critical foundation for future RCTs aimed at establishing robust clinical evidence for zinc supplementation in patients with CKD and those undergoing hemodialysis.

## Figures and Tables

**Table 1:** Summary of RCTs of zinc supplementation in patients with hemodialysis.

Author, Year [Reference]	Number of Subjects	Age (Years) ^†^	Elemental Zinc Dose (mg/day)	Administration Duration (Days)	Outcomes
Haddadian-Khouzani S et al., 2022 [15]	44	48.1 ± 14.8	NA (30 mg of zinc gluconate)	84	Improve: sleep quality and albumin Not significant: CRP and QOL
Hosseibi R et al., 2022 [14]	46	54.1 ± 5.4	50	56	Increase: serum zinc, BMI Decrease: hs-CRP, BUN, FBG
Okamoto T et al., 2019 [16]	91	68	34 (polaprezinc) /50 (zinc acetate hydrate)	180	Increase: serum zinc Decrease: serum copper (zinc acetate hydrate vs. polaprezinc)
Escobedo-Monge et al., 2019 [17]	48 (children)	12.8 ± 4	15/30	365	Increase: BMI (30 mg/day group only)
Tonelli et al., 2015 [19]	150	62	25 and 50	90 and 180	Not significant
Kobayashi et al., 2015 [21]	70	69 ± 10	34	90/180/270/360	Increase: serum zinc Decrease: serum copper, ferritin
El-Shazly et al., 2015 [22]	30	13.2 ± 2.1	16.5	90	Increase: serum zinc, BMI Decrease: serum leptin
Argani et al., 2014 [23]	60	(50, 60)	90	60	Increase: serum zinc, albumin, hemoglobin, BMI Decrease: serum leptin
Pakfetrat et al., 2013 [24]	97	51.6 ± 16.8	50	43	Increase: serum zinc Decrease: homocysteine
Mazani et al., 2013 [25]	65	52.7 ± 12.6	100	60	Increase: serum zinc, GSH, MDA, SOD, TAC
Guo and Wang, 2013 [26]	65	59.7 ± 9.2	11	56	Increase: plasma zinc, albumin, hemoglobin, hematocrit, nPNA, SOD, vitamin C, vitamin E, CD4, D19 Decrease: plasma copper, CRP, MDA INF-b, TNF-*α*,
Rahimi-Ardabili et al., 2012 [27]	60	52.7 ± 12.7	100	60	Increase: Apo-AI, HDL-C, PON
Roozbeh et al., 2009 [28]	53	55.7	45	42	Increase: serum zinc, TC, HDL-C, LDL-C, TG
Rashidi et al., 2009 [29]	55	57.6	45	42	Increase: serum zinc
Nava-Hernandez and Amato, 2005 [30]	25	16.6	100	90	NA
Matson et al., 2003 [31]	15	60 (31, 76)	45	42	Not significant
Chevalier et al., 2002 [32]	27	51.9	50	40/90/90	Increase: serum zinc, LDL-C
Candan et al., 2002 [33]	34	45.6 (28, 64)	20	90	Increase: serum zinc Decrease: lipid peroxidation osmotic fragility
Jern et al., 2000 [34]	14	56.5 (23, 80)	45	40/90	Increase: serum zinc, nPNA
Brodersen et al., 1995 [35]	40	60	60	112	Increase: serum zinc

Note. ^†^ Age is shown as mean, mean ± standard deviation, or mean (lower limit, upper limit). Abbreviations: Apo-AI, apolipoprotein AI; BMI, body mass index; BUN, blood urea nitrogen; Ccr, creatinine clearance rate; CRP, C-reactive protein; ESA, erythropoiesis-stimulating agent; ERI, ESA resistance index; FBG, fasting blood glucose; GFR, glomerular filtration rate; GSH, whole blood glutathione peroxidase; HDL-C, high-density lipoprotein cholesterol; IL, interleukin; LDL-C, low-density lipoprotein cholesterol; MDA, malondialdehyde; NA, not available; nPNA, normalized protein equivalent of nitrogen appearance; PON, paraoxonase; QOL, quality of life; SOD, superoxide dismutase; TAC, total antioxidant capacity; TC, total cholesterol; TG, triglyceride; TNF, tumor necrosis factor.

**Table 2:** Summary of observational studies regarding the association between serum zinc levels or zinc intake and mortality in patients with hemodialysis.

Author, Year [Reference]	Number of Subjects	Number of Deaths (Follow-Up Period)	Conclusions
Yang CY, et al., 2012 [43]	Prevalent patients on maintenance hemodialysis (*n* = 43) and peritoneal dialysis (*n* = 68)	14 deaths (2 years)	Old age, hypoalbuminemia, and zinc deficiency were independent predictors of mortality.
Tonelli M, et al., 2018 [49]	Incident hemodialysis (*n* = 1278)	260 deaths (2 years)	Lower level of zinc was not associated with higher risk of death.
Toida T, et al., 2020 [5]	Incident hemodialysis (*n* = 142)	15 deaths (2.5 years)	The association between serum zinc levels and all-cause mortality was not clear after adjustments for potential confounders.
Knehtl M, et al., 2022 [142]	Prevalent patients on maintenance hemodialysis (*n* = 61)	11 deaths (2.8 years)	No significant association of serum zinc with mortality.
Nakatani S, et al., 2024 [144]	Prevalent patients on Maintenance hemodialysis (*n* = 1662)	468 deaths (5 years)	A lower serum zinc level was a significant factor predicting a higher risk of mortality in those with lower serum albumin.
Garagarza C, et al., 2022 [145]	Prevalent patients on Maintenance hemodialysis (*n* = 582)	29 deaths (1 year)	Lower zinc intake below was a significant factor predicting a higher risk of mortality.

## Abbreviations

The following abbreviations are used in this manuscript:

AAC	Abdominal aortic calcification
ALP	Alkaline Phosphatase
BMI	Body mass index
BMSCs	Bone Marrow-Derived Mesenchymal Stem Cells
CIMT	Carotid intima-media thickness
CKD	Chronic kidney disease
eGFR	Estimated glomerular filtration rate
CPP	Calciprotein particle
CRP	C-reactive protein
CVD	Cardiovascular disease
ESA	Erythropoiesis-stimulating agent
HbA1c	Glycosylated hemoglobin
HIF	Hypoxia-inducible factor
HIF-PHIs	Hypoxia-Inducible Factor Prolyl Hydroxylase Inhibitors
HOMAIR	Homeostasis model assessment-estimated insulin resistance
Nrf2	Nuclear Factor Erythroid 2–Related Factor 2
NF-κB	nuclear factor kappa light chain enhancer of activated B
NO	Nitric Oxide
NOS	Nitric Oxide Synthase
OCN	Osteocalcin
PWV	Pulse wave velocity
RCT	Randomized controlled trial
RUNX2	Runt-Related Transcription Factor 2
SOD	Superoxide Dismutase
SOX9	SRY-Box Transcription Factor 9
T50	Serum calcification propensity
TNF	Tumor necrosis factor
TNFAIP3	TNFα-induced protein 3
VSMC	vasculature smooth muscle cell
